# Genome editing in rice mediated by miniature size Cas nuclease SpCas12f

**DOI:** 10.3389/fgeed.2023.1138843

**Published:** 2023-03-13

**Authors:** Satoru Sukegawa, Osamu Nureki, Seiichi Toki, Hiroaki Saika

**Affiliations:** ^1^ Division of Crop Genome Editing Research, Institute of Agrobiological Sciences, National Agriculture and Food Research Organization, Tsukuba, Ibaraki, Japan; ^2^ Department of Biological Sciences, Graduate School of Science, The University of Tokyo, Bunkyo-ku, Tokyo, Japan; ^3^ Graduate School of Nanobioscience, Yokohama City University, Yokohama, Kanagawa, Japan; ^4^ Kihara Institute for Biological Research, Yokohama City University, Yokohama, Kanagawa, Japan; ^5^ Department of Plant Life Science, Faculty of Agriculture, Ryukoku University, Otsu, Shiga, Japan

**Keywords:** CRISPR-Cas12f, deletion, genome editing, Oryza sativa, targeted mutagenesis, syntrophomonas palmitatica, transformation

## Abstract

Cas9 derived from *Streptococcus pyogenes* (SpCas9) is used widely in genome editing using the CRISPR-Cas system due to its high activity, but is a relatively large molecule (1,368 amino acid (a.a.) residues). Recently, targeted mutagenesis in human cells and maize using Cas12f derived from *Syntrophomonas palmitatica* (SpCas12f)—a very small Cas of 497 a.a, which is a more suitable size for virus vectors—was reported. However, there are no reports of genome editing using SpCas12f in crops other than maize. In this study, we applied SpCas12f to genome editing in rice—one of the most important staple crops in the world. An expression vector encoding rice codon-optimized SpCas12f and sgRNA for *OsTubulin* as a target was introduced into rice calli by *Agrobacterium*-mediated transformation. Molecular analysis of SpCas12f-transformed calli showed that mutations were introduced successfully into the target region. Detailed analysis by amplicon sequencing revealed estimated mutation frequencies (a ratio of the number of mutated calli to that of SpCas12f-transformed calli) of 28.8% and 55.6% in two targets. Most mutation patterns were deletions, but base substitutions and insertions were also confirmed at low frequency. Moreover, off-target mutations by SpCas12f were not found. Furthermore, mutant plants were regenerated successfully from the mutated calli. It was confirmed that the mutations in the regenerated plants were inherited to the next-generation. In the previous report in maize, mutations were introduced by treatment with heat shock at 45°C for 4 h per day for 3 days; no mutations were introduced under normal growth conditions at 28°C. Surprisingly, however, mutations can be introduced without heat-shock treatment in rice. This might be due to the culture conditions, with relatively higher temperature (30°C or higher) and constant light during callus proliferation. Taken together, we demonstrated that SpCas12f can be used to achieve targeted mutagenesis in rice. SpCas12f is thus a useful tool for genome editing in rice and is suitable for virus vector-mediated genome editing due to its very small size.

## 1 Introduction

Genome editing technology using the clustered regularly interspaced short palindromic repeats (CRISPR)-CRISPR-associated proteins (Cas) system can be used for gene knock-out, insertion of a target sequence, and regulation of gene expression very easily and rapidly in various species including mammals, plants, insects and fishes (Doudna and Charpentier, 2014). The CRISPR-Cas system is an adaptive immune system present in some bacteria to reject various parasites and infections (Sorek et al., 2013). When a phage infects a bacterium, CRISPR RNA (crRNA) interacts with trans-activating CRISPR RNA (tracrRNA) to form a guide RNA (gRNA), and Cas nuclease binds to that gRNA to form a gRNA-CRISPR complex. This RNA-protein complex accesses and binds to the target sequence on the DNA of the infected phage based on the target sequence specified in the crRNA using the Protospacer Adjacent Motif (PAM) sequence as a guide, and cleaves the target sequence, thus inactivating the infecting organism (Jiang and Doudna, 2017).

Genome editing using the CRISPR-Cas system applies this bacterial defense system to cleave target DNA sequences with Cas nuclease and introduces mutations due to errors in the subsequent DNA repair pathway. *Agrobacterium*-mediated transformation is used widely to deliver the CRISPR-Cas9 system into plants. Exogenous DNA harboring Cas9 and a single gRNA (sgRNA) expression cassette as a transgene is first introduced into the host plant genome. Genome-edited plants without the transgene are obtained in the progeny after segregation. However, it is difficult to apply this approach to all plants due to, for example, vegetative propagation and high heterozygosity. Recently, some CRISPR-Cas9 delivery systems that avoid the need to insert exogenous DNA into the host genome have been developed. Virus vector-mediated CRISPR-Cas9 delivery is one such approach to transgene-free genome editing. There are some reports of successful targeted mutagenesis in plants by delivery of Cas protein using a virus vector (Cody and Scholthof, 2019). However, there is a negative correlation between insert length and stability, and limits to the length of inserts in virus vectors (Gleba et al., 2004; Avesani et al., 2007). Although it is the most commonly used Cas nuclease because it can cleave double-stranded DNA efficiently, *Streptococcus pyogenes*-derived Cas9 (SpCas9) has a large molecular weight and is 1,368 amino acids (a.a.) long. Thus, SpCas9-mediated genome editing *via* a virus vector can be inefficient due to this large size.

In recent years, there have been many reports of genome editing using Cas proteins smaller than SpCas9. Cas9, which is derived from *Staphylococcus aureus* (SaCas9, 1,053 a.a.), recognizes 5′-NNGRRT-3′ (R = A/G) as a PAM sequence and can induce mutations in plants such as tobacco, Arabidopsis, rice, and citrus (Steinert et al., 2015; Kaya et al., 2016; Jia et al., 2017; Qin et al., 2019). Also, Cas9 derived from *Campylobacter jejuni* (CjCas9, 984 a.a.), which recognizes 5′-NNNVRYAC-3′ (V = A/G/C, Y = T/C) as a PAM sequence, is even smaller than SaCas9 and can be delivered *via* a viral vector, adeno-associated virus (AAV), to induce targeted mutagenesis in human and mouse cells (Kim et al., 2017a; Yamada et al., 2017; Nakagawa et al., 2022). Of the several Cas nucleases that belong to a class other than Cas9, Cas12a (a type V Cas nuclease) is smaller than, or similar in size to, SpCas9 (750–1,373 a.a.) and can be applied to genome editing in various plants including Arabidopsis, rice, tobacco, soybean, and tomato (Endo et al., 2016; Kim et al., 2017b; Tang et al., 2017; Wang et al., 2017; Yin et al., 2017; Jia et al., 2019; Schindele and Puchta, 2020; Vu et al., 2020). Cas12b, also smaller than Cas nuclease (AaCas12b: 1,129 a.a.), recognizes a T-rich PAM similar to Cas12a (Teng et al., 2018; Strecker et al., 2019). Cas12b can induce mutation in rice and cotton (Ming et al., 2020; Wang et al., 2020). Cas12j, which, at 700–800 a.a, is even smaller than the above Cas nucleases, actively cleaves target sequences in HEK293 cells and Arabidopsis protoplasts (Pausch et al., 2020).

Among the miniature-size Cas nucleases that have been reported, in this study we focused on Cas12f—a type V Cas system with a very small nuclease (422–603 a.a.). Cas12f, which is derived from uncultured archaeon (Un1Cas12f1), was initially reported to have a single-strand DNA cleaving activity (Harrington et al., 2018). Recently, it was reported to also cleave double-stranded DNA (Karvelis et al., 2020). Cas12f recognizes a T-rich PAM sequence similar to other type V Cas nucleases. For example, Un1Cas12f1 cleaves the 20th to the 22nd base from the 3′ of the PAM sequence on the non-targeted strand (NTS) and the 24th base from the PAM on the targeted strand (TS). Cas12f derived from *Syntrophomonas palmitatica* (SpCas12f) cleaved the NTS and TS at 24th and 22nd base from the PAM (5′-TTC-3′), respectively (Karvelis et al., 2020). Unlike other Cas nucleases, Cas12f forms an asymmetric dimer (Takeda et al., 2021; Xiao et al., 2021). This homodimer recognizes the target sequence by forming a complex with sgRNA, and a single RuvC domain cleaves both strands of the target double-stranded DNA (Takeda et al., 2021; Xiao et al., 2021). Since Un1Cas12f1 has low activity in mammalian cells, it has been reported that its activity can be increased by modifying the amino acid sequence of Un1Cas12f1 or designing a specific sgRNA (Xu et al., 2021; Kim et al., 2022). Cas12f derived from *Acidibacillus sulfuroxidans* (AsCas12f1) also has double-strand DNA cleaving activity and can induce mutation in bacterial and human cells (Wang et al., 2021; Wu et al., 2021). Just recently, it has been reported that targeted mutagenesis by SpCas12f was introduced successfully in maize by performing multiple heat shock treatments at 45°C (Bigelyte et al., 2021).

In this study, our aim was to obtain more basic information about CIRSPR-Cas12f-mediated genome editing in plants. To do this, we demonstrate targeted mutagenesis using Cas12f in rice callus. Following expression of SpCas12f and sgRNA targeting rice *α-Tubulin* (LOC_Os03g51600) in rice callus derived from mature seeds, some mutations, mainly deletions, were introduced successfully at the target site. Furthermore, using regenerated plants obtained from calli harboring mutations at the target site, we were able to confirm that these mutations were inherited to next-generation plants. These results suggest that, like other Cas nucleases, miniature Cas12f can be used as a genome editing tool for basic research and breeding in rice and might be applicable to virus vector-mediated genome editing in plants.

## 2 Materials and methods

### 2.1 Oligonucleotides

The oligonucleotides used in this study are listed in Supplementary Table S1.

### 2.2 Vector construction

**FIGURE 1:**
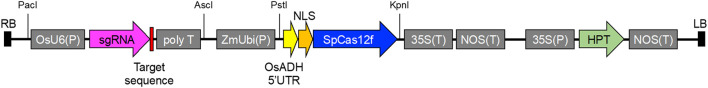
Vector construction of pPZP-SpCas12f Tub-1 and pPZP-SpCas12f Tub-2. Vector carrying sgRNA for OsTub-1 or OsTub-2 under the control of the rice U6 promoter OsU6(P), rice-codon optimized NLS-SpCas12f gene under the control of the maize ubiquitin 1 promoter ZmUbi(P), and the hygromycin phosphotransferase gene under the control of the cauliflower mosaic virus 35S promoter 35S(P) as a selection marker. To enhance SpCas12f translation, the 5′ untranslated region of rice alcohol dehydrogenase (OsADH) was used. LB and RB; left and right borders of T-DNA, respectively; NLS, nuclear localization signal; 35S(T), cauliflower mosaic virus 35S terminator; NOS(T), nopaline synthase terminator; HPT, hygromycin phosphotransferase.

Information on the SpCas12f protein and sgRNA sequences was obtained from a previous study (Bigelyte et al., 2021). DNA fragments of rice-codon-optimized SpCas12f including a nuclear localization signal (NLS) at the N terminus with the 5′ untranslated region of rice alcohol dehydrogenase and sgRNA including an OsU6 promoter were synthesized by GeneArt Gene Synthesis (Thermo Fisher Scientific). The synthesized DNA fragment of SpCas12f was inserted into *Pst*I/*Kpn*I-digested pPZP ZmUbi-SpCas9-NG vector harboring SpCas9-NG and an HPT expression cassette by an In-fusion reaction (Takara), yielding an SpCas12f expression vector, pPZP-SpCas12f (Supplementary Table S2). The synthesized DNA fragment of sgRNA was inserted into the *Asc*I/*Pac*I site of the pUCAP vector (van Engelen et al., 1995) by ligation reaction with T4 DNA ligase (New England BioLabs), yielding pUCAP-sgRNA (Supplementary Table S2). The annealed oligonucleotide pairs for the target sequences were cloned into the *Bbs*I site in pUCAP-sgRNA vector by ligation reaction. The resultant sgRNA expression cassette containing the target sequence was digested with *Asc*I/*Pac*I and inserted into the *Asc*I/*Pac*I site in the pPZP-SpCas12f vector by ligation reaction, yielding pPZP-SpCas12f Tub-1 and pPZP-SpCas12f Tub-2 (Figure 1).

### 2.3 Rice transformation and regeneration

Binary vectors, pPZP-SpCas12f Tub-1 and pPZP-SpCas12f Tub-2 were introduced into *Agrobacterium* EHA105 strain (Hood et al., 1993) by electroporation. The transformation was conducted following a previously published protocol (Toki, 1997). Callus was induced by culturing mature seeds on N6D medium at 30°C under constant light. After a 4-week cultivation, calli were infected with *Agrobacterium* harboring a binary vector and co-cultivated for 3 days on 2N6AS medium at 23°C in the dark. Infected calli were treated with sterilized water containing 50 mg/L meropenem (FUJIFILM) and cultured on N6D medium including 50 mg/L hygromycin (FUJIFILM) and 25 mg/L meropenem at 30°C under constant light. Transformed calli were cultured on ReIII medium for 3–4 weeks at 27°C under 16 h light/8 h dark. The regenerated plants obtained were transferred to MS/hormone-free medium and cultured for 2 weeks at 27°C under 16 h light/8 h dark. Finally, the rice seedlings were transferred to soil and grown in a greenhouse until seeds were produced.

### 2.4 Heteroduplex mobility assay and sanger sequence analysis

For molecular analysis, hygromycin-resistant calli after 5–6 weeks of selection were regenerated into whole plants and T_1_ seeds were harvested. Genomic DNA was extracted from calli and leaves as described by Edwards et al. (1991). PCR was conducted using KOD One PCR Master Mix (Toyobo). The detailed experimental method followed the manufacturer’s protocols. For heteroduplex mobility assay (HMA), PCR products were diluted 15-fold. Diluted PCR products were subjected to electrophoresis on a microchip electrophoresis device, MultiNA (Shimadzu). Images were obtained following the manufacturer’s protocol.

For Sanger sequence analysis, PCR products were cloned into pCR-BluntII-TOPO using a Zero Blunt TOPO PCR Cloning Kit (Thermo Fisher Scientific). The resultant vectors were transformed into *E. coli* and colony PCR conducted to amplify the DNA fragments with M13 Forward/M13 Reverse primers and KOD One PCR Master Mix. The Sanger sequencing reaction used a BigDye Terminator v3.1 Cycle Sequencing Kit (Thermo Fisher Scientific) and the detailed method followed the manufacturer’s protocols. Sequencing reaction products were purified using a BigDye XTerminator Purification Kit (Thermo Fisher Scientific) following the manufacturer’s protocols and capillary electrophoresis was run on 3,500/3500xL Genetic Analyzer (Thermo Fisher Scientific). Data were analyzed with the Snapgene (GSL Biotech).

### 2.5 Amplicon sequence analysis

The first PCR was conducted with KOD One PCR Master Mix. PCR fragments of approximately 400 bp were used for amplicon sequencing on an Illumina MiSeq platform (performed by Seibutsu Giken). Amplicons sequence results were analyzed for mutations using CRISPResso2 (Clement et al. (2019), http://crispresso2.pinellolab.org). Mutation patterns detected at a rate lower 0.2% of the total number of reads were excluded from analysis.

## 3 Results

### 3.1 Targeted mutagenesis of *OsTubulin* in rice calli by SpCas12f

**FIGURE 2:**
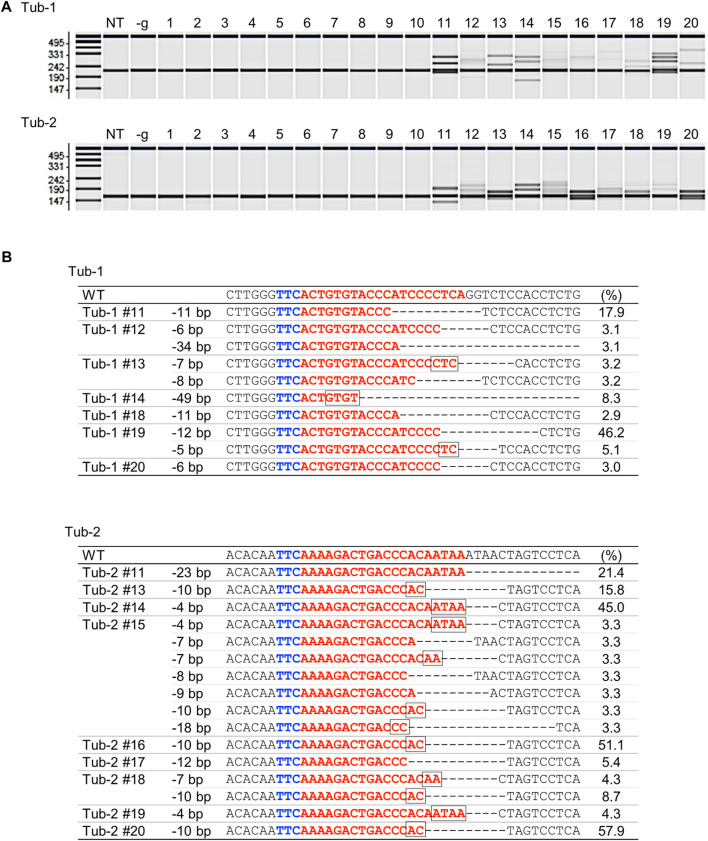
Analysis of SpCas12f-induced mutations in rice calli **(A)** Heteroduplex mobility assay (HMA) to detect mutations on Tub-1 (upper panel) and Tub-2 (lower panel) in transformed calli. We picked up 10 HMA-negative samples and 10 HMA-positive samples from Supplementary Figure S1. The numbers 1–10 and 11–20 indicate representative HMA-negative and -positive samples, respectively. NT; non-transformed, -g; calli transformed with pPZP-SpCas12f vector without sgRNA. **(B)** Examples of Sanger sequencing results in HMA positive samples on Tub-1 (upper panel) and Tub-2 (lower panel) in transformed calli. Blue and red letters on the sequence indicate the PAM sequence and the target sequence, respectively. The square box on the sequence indicates microhomology sequences located at the breakpoint. The number shown to the right side of each sequence is the frequency (ratio of the number of clones in which mutations were detected to total number of clones analyzed). The remaining percentage in each callus is the frequency of WT (no mutations). PCR products from pPZP-SpCas12f Tub-1 or pPZP-SpCas12f Tub-2 transformed calli were cloned in *E. coli*, and 19–45 clones were sequenced and analyzed.—indicates deletion.

To verify whether targeted mutagenesis could be achieved in rice using SpCas12f, expression vectors harboring SpCas12f driven by the maize ubiquitin promoter and sgRNA including a 20-nt target sequence for rice *α-tubulin* (Tub-1 or Tub-2) were constructed (Figure 1; Table 1). These binary vectors were transformed into rice calli *via Agrobacterium*. HMA performed using PCR fragments amplified from genomic DNA extracted from transformed calli revealed multiple bands in some samples, although single bands of the same size as a non-transformed sample were found in others (Figure 2A), suggesting that mutations had been introduced into the target region by SpCas12f. The average frequencies of HMA-positive samples were 8.6% and 24.0% in Tub-1 and Tub-2 sites, respectively, in the three replicate experiments (Table 1). The mutation patterns of PCR products were analyzed by Sanger sequencing in 7 and 9 lines of HMA-positive calli transformed with pPZP-SpCas12f Tub-1 and pPZP-SpCas12f Tub-2, respectively. Sequencing analysis showed that various types of deletion were detected in the target regions (Figure 2B). In calli transformed with pPZP-SpCas12f Tub-1, short deletions of about five bp to slightly longer deletions of 49 bp were detected. Similarly, in calli transformed with pPZP-SpCas12f Tub-2, deletions of 4–23 bp were detected. No insertions or base substitutions were detected by Sanger sequence analysis. These results showed that mutations had been introduced successfully into the target region in rice using SpCas12f.

**TABLE 1:** Summary of HMA in SpCas12-transformed calli.

Target site*	Mutation rate (HMA positive/analyzed calli)	
OsTub-1 TTC ACTGTGTACCCATCCCCTCA	Experiment 1	14.6% (14/96)
	Experiment 2	4.8% (3/62)
	Experiment 3	6.3% (1/16)
	average ± s.d	8.6% ± 3.0%
OsTub-2 TTC AAAAGACTGACCCACAATAA	Experiment 1	31.3% (15/48)
	Experiment 2	15.6% (30/192)
	Experiment 3	25.0% (24/96)
	average ± s.d	24.0% ± 4.5%

*Blue and red letters indicate the PAM, sequence and the target sequence, respectively.

**FIGURE 3:**
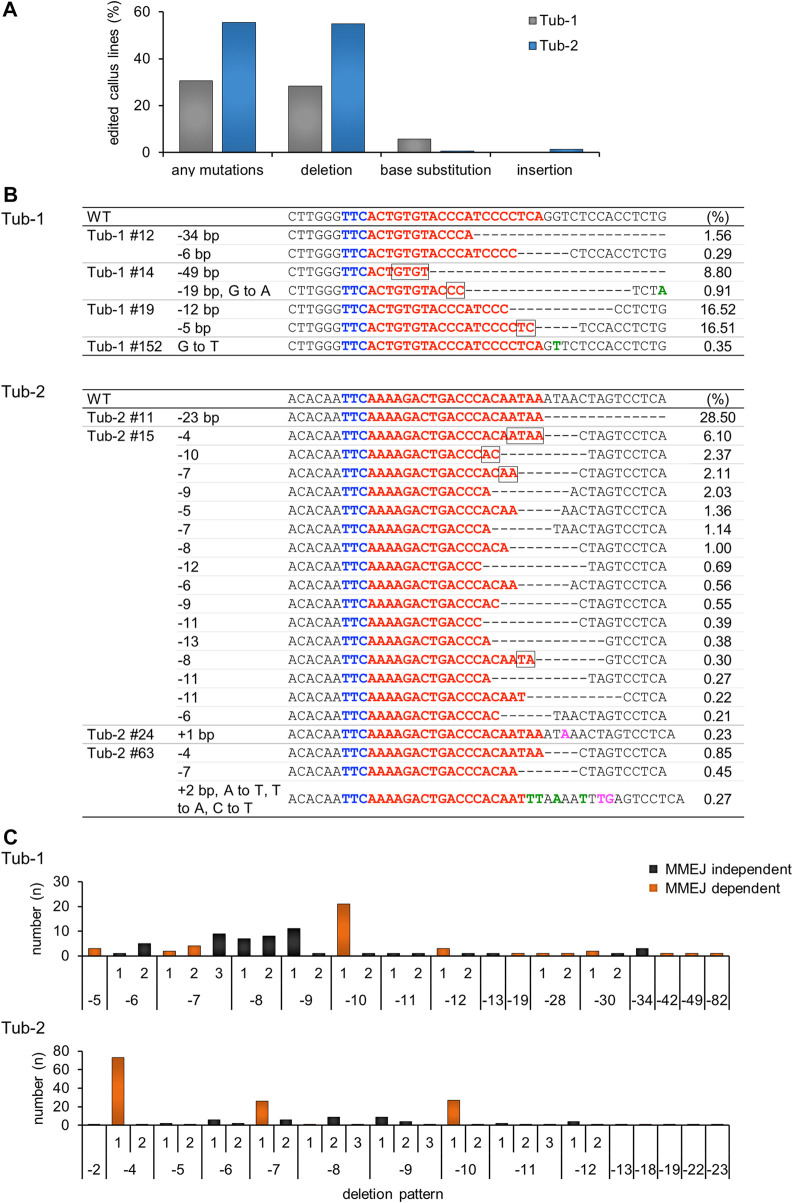
Amplicon sequence analysis in SpCas12f-transformed calli. **(A)** The percentage of transgenic callus lines in which mutations were introduction per total number of callus lines analyzed by amplicon sequencing is shown. The numbers represent the sum of calli analyzed in three replicate experiments. Individual mutation patterns are classified into deletion, base substitution, and base insertion, and the ratio of each mutation pattern is also shown. **(B)** Examples of amplicon sequence results on Tub-1 (upper panel) and Tub-2 (lower panel) in transformed calli. Details are the same as in Figure 2B and green or magenta letters on the sequence indicate base substitution or base insertion, respectively. The number shown to the right of each sequence is the frequency, i.e., the ratio of the number of amplicon sequence reads. **(C)** Histogram showing the correlation between the deletion pattern and the number of samples on Tub-1 (upper panel), and Tub-2 (lower panel). The horizontal axis shows the deletion pattern shown in Supplementary Table S3, and the vertical axis shows the number of samples for which the deletion pattern was detected. The numbers are the sum of calli analyzed in three replicate experiments. Black bars, MMEJ independent deletion pattern; orange bars, deletions caused by MMEJ.

To analyze the mutation pattern introduced by SpCas12f in more detail, we performed amplicon sequencing analysis, which revealed that the average mutation efficiency in the Tub-1 and Tub-2 sites, i.e., the ratio of number of mutated calli to total analyzed calli, was 28.8% and 55.6%, respectively, in three independent replicate experiments (Figure 3A), whereas no mutations were observed in the negative control (SpCas12f-transformed callus without sgRNA). Due to the low frequency, mutations were detected even in samples that were HMA-negative by amplicon sequence analysis. Interestingly, base substitution and single base insertion, which could not be detected in Sanger sequence analysis, were detected at extremely low frequency, and the most common mutation patterns were deletions (Figures 3A, B). Focusing on the deletion patterns in each sample, the number of deletion patterns derived from microhomology-mediated end-joining (MMEJ) repair was 12 and five in Tub-1 and Tub-2 sites, while the number of all deletion patterns were 26 and 27, respectively (Figure 3C; Supplementary Table S3). At the Tub-1 site, a MMEJ-dependent 10-bp deletion was most likely to occur, while MMEJ-independent eight- to 9-bp deletions occurred to a similar, albeit slightly lesser, extent. At the Tub-2 site, MMEJ-dependent 4-, 7-, and 10-bp deletions occurred very frequently, but less frequent MMEJ-independent deletion of two- to 23-bp also occurred (Figure 3C; Supplementary Table S3). No samples with off-target mutations introduced near the target sites were found (0/173 samples, Table 2; off-target analysis examined a two-base-mismatched target by amplicon sequence analysis in the same way as the Tub-1 target). Moreover, to demonstrate that mutations with SpCas12f could be introduced into a target other than the *OsTubulin* locus, targeted mutagenesis of the *OsHDAC1* (LOC_Os06g38470) locus was performed. Mutations were confirmed in transformed calli, although the mutation frequency was lower than that obtained with *OsTubulin* (Supplementary Figure S2).

**TABLE 2:** Off-target sequence of Tub-1.

	Sequence*	Mutation rate
Target for Tub-1	G TTC ACTGTGTACCCATCCCCTCA ​GGT​CTC​CAC​CTC​TGT​GGT​TGA​GCC	53/173
Off-target	G TTC AC G GTGTACCC G TCCCCTCA GGT​CTC​CAC​CTC​TGT​GGT​TGA​GCC	0/173

*Blue, red and green letters indicate the PAM, sequence, the target sequence, and mismatch sequences, respectively.

### 3.2 Regenerated plants and analysis of mutation in T_1_ plants

**FIGURE 4:**
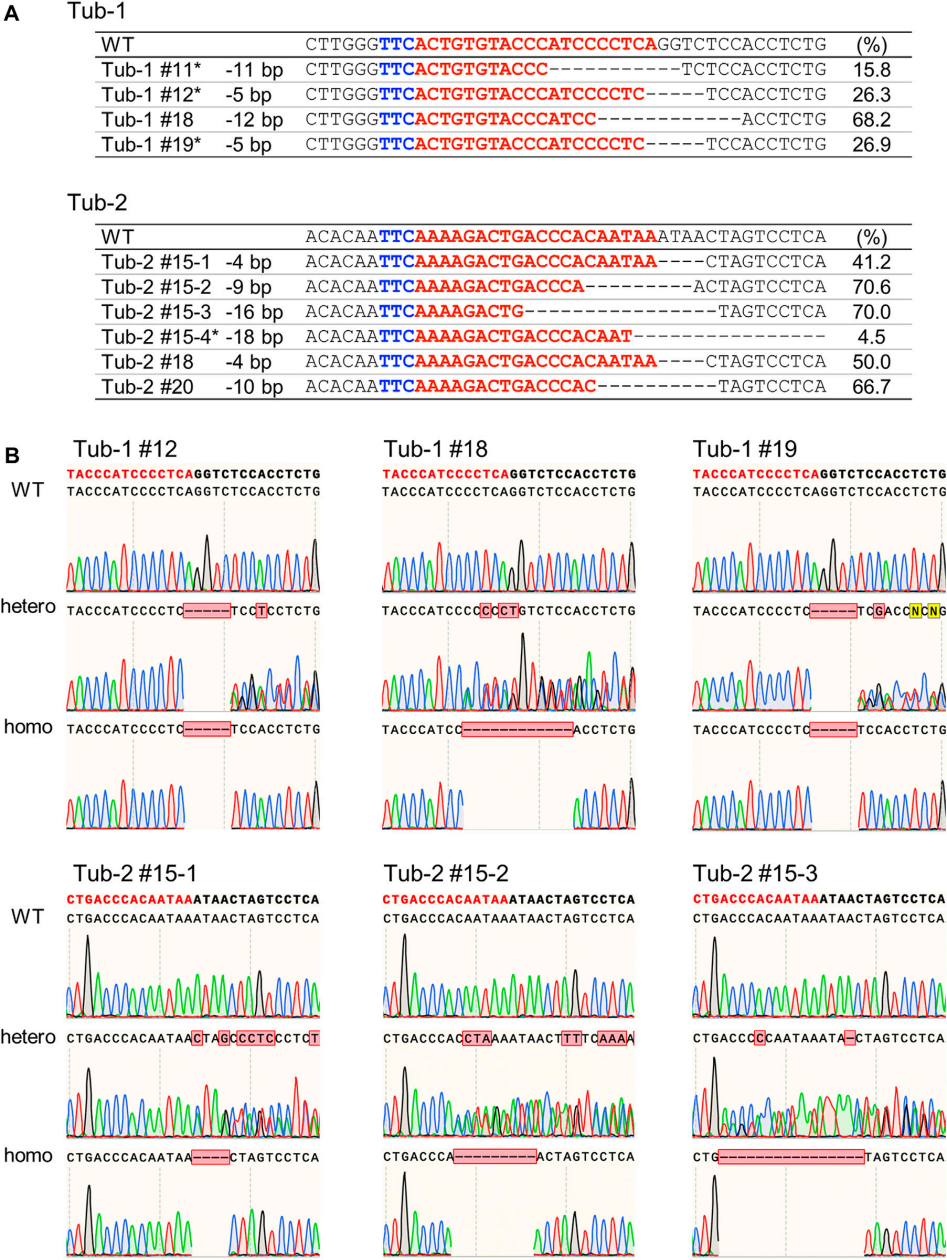
Analysis of SpCas12f-induced mutations in regenerated plants and their progenies in rice. **(A)** Sanger sequencing results on Tub-1 (upper panel) and Tub-2 (lower panel) in regenerated plants of Tub-1 (#11, #12, #18, #19, upper panel) and Tub-2 (#15, #18, #20, lower panel). Details as in Figure 2B. *The mutant: WT ratio did not fit to 1:1 by χ^2^-test. **(B)** Sequencing chromatograms at the Tub-1 and Tub-2 sites in T_1_ plants of Tub-1#12, Tub-1#18, Tub-1#19, Tub-2#15–1, Tub-2#15–2, and Tub-2#15–3. PCR fragments were sequenced directly.

Calli in which mutations by SpCas12f were detected were cultured on a regeneration medium to obtain regenerated plants. Genomic DNA extracted from the leaves of regenerated plants was analyzed by Sanger sequencing, confirming the presence of mutations in regenerated plants (Figure 4A). Deletions of 5–12 bp and 4–18 bp were induced in Tub-1 and Tub-2 sites in regenerated plants, respectively. Most of the mutation patterns in regenerated plants were the same as those detected in callus, but different mutation patterns were detected in some lines (Tub-2#15–3, Tub-2#15–4, and Tub2#18), most probably because the pieces of calli transferred to the regeneration medium were not the same as those used for HMA and Sanger sequencing analysis. All mutations detected in regenerated plants were mono-allelic and some regenerated plants could be mosaic in this experiment. These plants were cultivated in a greenhouse to obtain T_1_ seeds. As a result of Sanger sequencing analysis in the leaves of T_1_ plants of Tub-1#12, Tub-1#18, Tub-1#19, Tub-2#15–1, Tub-2#15–2, and Tub2-#15–3, the same mutations observed in regenerated plants and their segregation were detected (Figure 4B). T_1_ plants in which WT or mutation were heterozygous or homozygous were obtained at a ratio of 3: 4: Three in Tub-1#12, 2: 5: Three in Tub-1#18, 3: 3: Four in Tub-1#19, 3: 4: Three in Tub-2#15–1, 2: 4: Four in Tub-2#15–2 and, 2: 4: Three in Tub-2#15–3. They fit a 1:2:1 ratio (*p* = 0.82, 0.90, 0.41, 0.82, 0.55, and 0.85, respectively), suggesting that the mutations were inherited stably in a Mendelian manner. No apparent differences in plant size or root elongation were found between WT and mutant homozygous plants (Supplementary Figure S3). In addition, as was the case in callus, no off-target mutations were confirmed in T_1_ plants (Tub-1#12) (Table 2; Supplementary Table S4). Taken together, it was demonstrated that mutations introduced in rice using SpCas12f can be inherited successfully to the next-generation.

## 4 Discussion

Cas12f is very small compared with other Cas proteins, which is a great advantage in genome editing using virus vectors, but more information about genome editing using this new tool is needed. In this study, we demonstrated the introduction of mutations in rice using SpCas12f. The mutation efficiency and mutation pattern in rice calli were analyzed as well as mutations in next-generation plants. We succeeded in achieving targeted mutagenesis in rice calli at the target site at frequencies of 28.8% and 55.6% using SpCas12f (Figure 3A). SpCas12f has been reported to bind and cleave double-stranded DNA efficiently under high-temperature conditions (45°C–55°C) *in vitro* (Bigelyte et al., 2021). Thus, it had been considered that heat shock treatment is essential to activate the DNA digestion activity of SpCas12f necessary to introduce mutations in plants at useful frequencies. Indeed, according to a recent report of targeted mutagenesis by SpCas12f in maize, mutations were introduced by treatment with a 4-h heat shock of 45°C per day for 3 days, whereas no mutations were introduced under normal conditions (Bigelyte et al., 2021). In that experiment, immature maize embryos were cultured at 28 °C in the dark for 9–10 days and mutation analysis was performed in immature embryos 3 days after the onset of biolistic transformation, and in regenerated plants (Bigelyte et al., 2021). However, in our current study, *Agrobacterium*-infected rice calli were cultivated on selection medium containing hygromycin and meropenem at 30°C under constant light, and mutation analysis was performed 5–6 weeks after transformation. Rice calli can grow rapidly and vigorously under our tissue culture conditions (described in Materials and Methods, Toki (1997)). The long period of culture under constant and relatively high temperature could explain why mutations could be introduced by SpCas12f without heat shock in rice.

There have been some previous studies on the relationship between temperature and mutagenesis efficiency by Cas12a *in vivo*. For example, the efficiency of mutagenesis using Cas12a derived from *Francisella novicida* (FnCas12a), *Lachnospiraceae bacterium* (LbCas12a), and *Acidaminococcus* sp. BV3L6 (AsCas12a) was improved when rice calli were cultured at a higher temperature (28°C–37°C) rather than in normal growth conditions. In addition, the efficiency of targeted mutagenesis by LbCas12a increased at temperatures of 29°C and 28°C in Arabidopsis and wheat, respectively (Malzahn et al., 2019). Just recently, it has been reported that the mutation frequency by FnCas12a was increased by 1–4 heat shock treatments at 37°C for 24 h after stratification in Arabidopsis (Blomme et al., 2022). AsCas12a is more sensitive to temperature than Cas12a derived from other species (e.g., LbCas12a and FnCas12a) and required higher temperature conditions (28°C–34°C) to improve DNA cleaving activity in plants and zebrafish (Moreno-Mateos et al., 2017; Malzahn et al., 2019). Thus, heat shock treatment can be an option to increase the activity of temperature-sensitive Cas nuclease. Cultivation under higher temperature conditions or a series of heat shock treatments may make it possible to greatly increase the efficiency of mutation introduction by SpCas12f in rice.

In this study, the most common mutation patterns found by Sanger and amplicon sequence analysis were deletions of several to dozens of basepairs (Figures 2, 3). This result is similar to a previous report of Un1Cas12f- and AsCas12f1-mediated genome editing in human cells (Xin et al., 2022). Moreover, various types of MMEJ-mediated deletion were also found (Figure 3C; Supplementary Table S3). Both Cas12a and Cas12f generate sticky DNA ends (Zetsche et al., 2015; Karvelis et al., 2020). When Cas generates sticky ends, longer deletions are more likely to occur than in the case of blunt ends (Bothmer et al., 2017), and targeted mutagenesis by Cas12f or Cas12a seems to lead to longer deletions than when Cas9 is used.

Virus-mediated genome editing enables targeted mutagenesis without integration of the CRISPR-Cas transgene into the host genome in plants (Cody and Scholthof, 2019). To date, there have been reports of targeted mutagenesis in *Nicotiana benthamiana* achieved by agroinfiltration of sonchus yellow net rhabdovirus (SYNV) vector harboring SpCas9 and sgRNA (Ma et al., 2020) and mechanical inoculation of potato virus X (PVX) vector harboring SpCas9 and sgRNA (Ariga et al., 2020). However, the exogenous genes inserted into the virus genome are typically deleted from the viral vector during replication, and stability of the vector can be affected by both the length of the exogenous genes and temperature (Qu et al., 2005; Avesani et al., 2007; Choi et al., 2019; Ariga et al., 2020). With its small size, Cas12f thus represents a promising candidate tool for virus-mediated genome editing. UnCas12f mutants or engineered sgRNA for Cas12f enhance the activity of UnCas12f (Xu et al., 2021; Kim et al., 2022). Just recently, it was reported that engineered sgRNA of SpCas12f enhances genome editing frequency in human cells to levels comparable to those of FnCas12a (Wang et al., 2022). We envisage that genome editing frequency in plants can be similarly enhanced by the use of engineered sgRNA and/or modified SpCas12f, which will be developed in the near future.

## Data Availability

The datasets presented in this study can be found in online repositories. The names of the repository/repositories and accession number(s) can be found below: https://ddbj.nig.ac.jp/resource/sra-submission/DRA015026.
