# Supplementary material for: Genome editing in rice mediated by miniature size Cas nuclease SpCas12f
Source: Front Genome Ed. 2023 Mar 13;5:1138843. doi: 10.3389/fgeed.2023.1138843 (PMC10040665; doi:10.3389/fgeed.2023.1138843)

Supplementary Material

**Supplementary Table S1.** **Oligonucleotides used in this study**

|  | 5'-3' |
| --- | --- |
| sgRNA oligos for OsTub-1 | AAACACTGTGTACCCATCCCCTCA |
|  | AAAATGAGGGGATGGGTACACAGT |
| sgRNA oligos for OsTub-2 | AAACAAAAGACTGACCCACAATAA |
|  | AAAATTATTGTGGGTCAGTCTTTT |
| sgRNA oligos for OsHDAC | AAACCCCCCGCCTCCGGCGGAGGC |
|  | AAAAGCCTCCGCCGGAGGCGGGGG |
| HMA for OsTub-1 | GTCTCCAAGGTTTCCTTGTGTT |
|  | AGCAGAACAGCAACATCAGTGT |
| HMA for OsTub-2 | TGACCATCAGCCTGAAAGGA |
|  | TGCCGATGTACAGCATATTCA |
| HMA for OsHDAC | GGCAAAGAGGGGAAAAGAAA |
|  | CCTCCGCGTCGTAGAAGTAG |
| 1st PCR for amplicon sequence of OsTub-1 | TCGTCGGCAGCGTCAGATGTGTATAAGAGACAGTACAGTTGGCAAGGAGATTGTTGA |
|  | GTCTCGTGGGCTCGGAGATGTGTATAAGAGACAGtACCTGAGACACAAGCCTGTTGAG |
| 1st PCR for amplicon sequence of OsTub-2 | TCGTCGGCAGCGTCAGATGTGTATAAGAGACAGGTGGAACAAATACTCCACGCATGTA |
|  | GTCTCGTGGGCTCGGAGATGTGTATAAGAGACAGACAAATACAGCACGGGGAACA |

**Supplementary Table S2. T-DNA sequences used in this study**

GTTTACCCGCCAATATATCCTGTCAAACACTGATAGTTTAAACTGAAGGCGGGAAACGACAATCTGATCCTGGCGAAAGGGGGATGTGCTGCAAGGCGATTAAGTTGGGTAACGCCAGGGTTTTCCCAGTCACGACGTTGTAAAACGACGGCCAGTGCCAAGCTcttaatTAAGAGCTCGGATCCACTAGTAACGGCCGCCAGTGTGCTGGAATTGCCCTTGGATCATGAACCAACGGCCTGGCTGTATTTGGTGGTTGTGTAGGGAGATGGGGAGAAGAAAAGCCCGATTCTCTTCGCTGTGATGGGCTGGATGCATGCGGGGGAGCGGGAGGCCCAAGTACGTGCACGGTGAGCGGCCCACAGGGCGAGTGTGAGCGCGAGAGGCGGGAGGAACAGTTTAGTACCACATTGCCCAGCTAACTCGAACGCGACCAACTTATAAACCCGCGCGCTGTCGCTTGTGTTATTTACTCTGTTTCGCGCGCCAGGGCAGTTAGGTGCCCTAAAAGAGCGAAGTGGCCGAAAGGAAAGGCTAACGCTTCTCTAACGCTACGGCGACCTTGGCGAAATGCCATCAATACCACGCGGCCCGAAAGGGTTCGCGCGAAACTGAGTAATGAAAGTCGCATCTTGCGTAAGCGCGTGGATTGAAACACTGTGTACCCATCCCCTCATTTTTTTGTCCCTTCGAAGGGCAATTCTGCAGATATCCATCACACTGTCGAGGTCGACGGTATCGATAAGCTTGGcgcgccaagcttTAGAGATAATGAGCATTGCATGTCTAAGTTATAAAAAATTACCACATATTTTTTTTGTCACACTTGTTTGAAGTGCAGTTTATCTATCTTTATACATATATTTAAACTTTACTCTACGAATAATATAATCTATAGTACTACAATAATATCAGTGTTTTAGAGAATCATATAAATGAACAGTTAGACATGGTCTAAAGGACAATTGAGTATTTTGACAACAGGACTCTACAGTTTTATCTTTTTAGTGTGCATGTGTTCTCCTTTTTTTTTGCAAATAGCTTCACCTATATAATACTTCATCCATTTTATTAGTACATCCATTTAGGGTTTAGGGTTAATGGTTTTTATAGACTAATTTTTTTAGTACATCTATTTTATTCTATTTTAGCCTCTAAATTAAGAAAACTAAAACTCTATTTTAGTTTTTTTATTTAATAATTTAGATATAAAATAGAATAAAATAAAGTGACTAAAAATTAAACAAATACCCTTTAAGAAATTAAAAAAACTAAGGAAACATTTTTCTTGTTTCGAGTAGATAATGCCAGCCTGTTAAACGCCGTCGACGAGTCTAACGGACACCAACCAGCGAACCAGCAGCGTCGCGTCGGGCCAAGCGAAGCAGACGGCACGGCATCTCTGTCGCTGCCTCTGGACCCCTCTCGAGAGTTCCGCTCCACCGTTGGACTTGCTCCGCTGTCGGCATCCAGAAATTGCGTGGCGGAGCGGCAGACGTGAGCCGGCACGGCAGGCGGCCTCCTCCTCCTCTCACGGCACGGCAGCTACGGGGGATTCCTTTCCCACCGCTCCTTCGCTTTCCCTTCCTCGCCCGCCGTAATAAATAGACACCCCCTCCACACCCTCTTTCCCCAACCTCGTGTTGTTCGGAGCGCACACACACACAACCAGATCTCCCCCAAATCCACCCGTCGGCACCTCCGCTTCAAGGTACGCCGCTCGTCCTCCCCCCCCCCCCCTCTCTACCTTCTCTAGATCGGCGTTCCGGTCCATGGTTAGGGCCCGGTAGTTCTACTTCTGTTCATGTTTGTGTTAGATCCGTGTTTGTGTTAGATCCGTGCTGCTAGCGTTCGTACACGGATGCGACCTGTACGTCAGACACGTTCTGATTGCTAACTTGCCAGTGTTTCTCTTTGGGGAATCCTGGGATGGCTCTAGCCGTTCCGCAGACGGGATCGATTTCATGATTTTTTTTGTTTCGTTGCATAGGGTTTGGTTTGCCCTTTTCCTTTATTTCAATATATGCCGTGCACTTGTTTGTCGGGTCATCTTTTCATGCTTTTTTTTGTCTTGGTTGTGATGATGTGGTCTGGTTGGGCGGTCGTTCTAGATCGGAGTAGAATTCTGTTTCAAACTACCTGGTGGATTTATTAATTTTGGATCTGTATGTGTGTGCCATACATATTCATAGTTACGAATTGAAGATGATGGATGGAAATATCGATCTAGGATAGGTATACATGTTGATGCGGGTTTTACTGATGCATATACAGAGATGCTTTTTGTTCGCTTGGTTGTGATGATGTGGTGTGGTTGGGCGGTCGTTCATTCGTTCTAGATCGGAGTAGAATACTGTTTCAAACTACCTGGTGTATTTATTAATTTTGGAACTGTATGTGTGTGTCATACATCTTCATAGTTACGAGTTTAAGATGGATGGAAATATCGATCTAGGATAGGTATACATGTTGATGTGGGTTTTACTGATGCATATACATGATGGCATATGCAGCATCTATTCATATGCTCTAACCTTGAGTACCTATCTATTATAATAAACAAGTATGTTTTATAATTATTTTGATCTTGATATACTTGGATGATGGCATATGCAGCAGCTATATGTGGATTTTTTTAGCCCTGCCTTCATACGCTATTTATTTGCTTGGTACTGTTTCTTTTGTCGATGCTCACCCTGTTGTTTGGTGTTACTTCTGCAGGAATTCCAAGCAACGAACTGCGAGTGATTCAAGAAAAAAGAAAACCTGAGCTTTCGATCTCTACGGAGTGGTTTCTTGTTCTTTGAAAAAGAGGGGGATTAGTCGACATGAAGAGGACAGCCGACGGCTCTGAGTTCGAGTCCCCGAAGAAGAAGCGCAAGGTCATGGGCGAGTCCGTGAAGGCCATCAAGCTCAAGATCCTCGACATGTTCCTCGATCCAGAGTGCACCAAGCAGGACGACAACTGGCGCAAGGACCTCTCCACCATGTCTAGGTTCTGCGCCGAGGCCGGCAATATGTGCCTCAGGGACCTCTACAACTACTTCTCCATGCCGAAAGAGGACCGCATCTCCTCCAAGGACCTGTACAACGCCATGTACCACAAGACCAAGCTCCTCCATCCTGAGCTGCCAGGCAAGGTGGCCAACCAGATCGTGAACCACGCCAAGGACGTGTGGAAGCGCAACGCCAAGCTCATCTACCGGAACCAGATCAGCATGCCGACCTACAAGATCACCACCGCGCCAATCCGCCTCCAGAACAACATCTACAAGCTGATCAAGAACAAAAACAAGTACATCATCGACGTGCAGCTCTACTCCAAAGAGTACAGCAAGGACTCCGGCAAGGGCACCCACCGCTACTTTCTCGTTGCCGTGAGGGATAGCAGCACCCGCATGATCTTCGACCGCATCATGAGCAAGGACCACATCGACTCCAGCAAGAGCTACACCCAGGGCCAGCTCCAGATCAAGAAGGACCACCAAGGCAAGTGGTACTGCATCATCCCGTACACGTTCCCGACGCACGAGACAGTGCTCGACCCGGATAAGGTGATGGGCGTTGACTTGGGCGTTGCCAAGGCCGTTTACTGGGCCTTCAACTCCTCCTACAAGCGCGGCTGCATTGACGGCGGCGAGATCGAGCACTTCCGCAAGATGATTAGAGCCCGCAGGGTGTCCATCCAGAATCAGATCAAACACTCCGGCGACGCCCGCAAAGGACATGGTAGAAAGAGAGCCCTCAAGCCGATCGAGACACTCTCCGAGAAAGAGAAGAACTTCCGCGACACCATCAACCACAGGTACGCCAACAGGATCGTCGAGGCCGCCATTAAGCAAGGCTGCGGCACCATCCAGATCGAGAATCTCGAGGGCATTGCCGACACCACCGGCTCCAAGTTCCTCAAGAACTGGCCGTACTACGACCTCCAGACCAAGATCGTCAACAAGGCCAAAGAACACGGCATCACCGTGGTGGCGATCAACCCACAGTACACCTCACAGCGCTGCTCCATGTGCGGCTACATCGAAAAGACCAACCGCTCCAGCCAGGCCGTGTTCGAGTGCAAGCAATGCGGCTATGGCTCCCGCACCATCTGCATCAACTGCAGGCATGTGCAAGTGAGCGGCGACGTGTGCGAAGAATGCGGCGGCATCGTGAAGAAAGAAAACGTGAACGCCGACTACAACGCCGCCAAGAACATCAGCACCCCGTACATCGATCAGATCATCATGGAAAAGTGCCTCGAGCTGGGCATCCCATACCGCTCCATCACGTGCAAAGAGTGCGGCCATATTCAGGCCTCCGGCAACACATGCGAAGTGTGCGGCTCCACCAACATCCTGAAGCCAAAGAAGATCCGCAAGGCCAAGTGAGGTACCGGAGGCTCTAAGAGGACCGCTGATGGCAGCGAGTTTGAGCCAAAGAAGAAGAGGAAGGTTTGAgagctcGATCTGTCGATCGACAAGCTCGAGTTTCTCCATAATAATGTGTGAGTAGTTCCCAGATAAGGGAATTAGGGTTCCTATAGGGTTTCGCTCATGTGTTGAGCATATAAGAAACCCTTAGTATGTATTTGTATTTGTAAAATACTTCTATCAATAAAATTTCTAATTCCTAAAACCAAAATCCAGTACTAAAATCCAGATCCCCCgaaGAATTTCCCCGATCGTTCAAACATTTGGCAATAAAGTTTCTTAAGATTGAATCCTGTTGCCGGTCTTGCGATGATTATCATATAATTTCTGTTGAATTACGTTAAGCATGTAATAATTAACATGTAATGCATGACGTTATTTATGAGATGGGTTTTTATGATTAGAGTCCCGCAATTATACATTTAATACGCGATAGAAAACAAAATATAGCGCGCAAACTAGGATAAATTATCGCGCGCGGTGTCATCTATGTTACTAGATCGGaattcgtaatcatggtcatagctgtttcctgtgggatccGTCCCCAGATTAGCCTTTTCAATTTCAGAAAGAATGCTAACCCACAGATGGTTAGAGAGGCTTACGCAGCAGGTCTCATCAAGACGATCTACCCGAGCAATAATCTCCAGGAAATCAAATACCTTCCCAAGAAGGTTAAAGATGCAGTCAAAAGATTCAGGACTAACTGCATCAAGAACACAGAGAAAGATATATTTCTCAAGATCAGAAGTACTATTCCAGTATGGACGATTCAAGGCTTGCTTCACAAACCAAGGCAAGTAATAGAGATTGGAGTCTCTAAAAAGGTAGTTCCCACTGAATCAAAGGCCATGGAGTCAAAGATTCAAATAGAGGACCTAACAGAACTCGCCGTAAAGACTGGCGAACAGTTCATACAGAGTCTCTTACGACTCAATGACAAGAAGAAAATCTTCGTCAACATGGTGGAGCACGACACACTTGTCTACTCCAAAAATATCAAAGATACAGTCTCAGAAGACCAAAGGGCAATTGAGACTTTTCAACAAAGGGTAATATCCGGAAACCTCCTCGGATTCCATTGCCCAGCTATCTGTCACTTTATTGTGAAGATAGTGGAAAAGGAAGGTGGCTCCTACAAATGCCATCATTGCGATAAAGGAAAGGCCATCGTTGAAGATGCCTCTGCCGACAGTGGTCCCAAAGATGGACCCCCACCCACGAGGAGCATCGTGGAAAAAGAAGACGTTCCAACCACGTCTTCAAAGCAAGTGGATTGATGTGATATCTCCACTGACGTAAGGGATGACGCACAATCCCACTATCCTTCGCAAGACCCTTCCTCTATATAAGGAAGTTCATTTCATTTGGAGAGAACACGGGGGAACTAGTATGAAAAAGCCTGAACTCACCGCGACGTCTGTCGAGAAGTTTCTGATCGAAAAGTTCGACAGCGTCTCCGACCTGATGCAGCTCTCGGAGGGCGAAGAATCTCGTGCTTTCAGCTTCGATGTAGGAGGGCGTGGATATGTCCTGCGGGTAAATAGCTGCGCCGATGGTTTCTACAAAGATCGTTATGTTTATCGGCACTTTGCATCGGCCGCGCTCCCGATTCCGGAAGTGCTTGACATTGGGGAGTTTAGCGAGAGCCTGACCTATTGCATCTCCCGCCGTTCACAGGGTGTCACGTTGCAAGACCTGCCTGAAACCGAACTGCCCGCTGTTCTACAACCGGTCGCGGAGGCTATGGATGCGATCGCTGCGGCCGATCTTAGCCAGACGAGCGGGTTCGGCCCATTCGGACCGCAAGGAATCGGTCAATACACTACATGGCGTGATTTtATATGCGCGATTGCTGATCCCCATGTGTATCACTGGCAAACTGTGATGGACGACACCGTCAGTGCGTCCGTCGCGCAGGCTCTCGATGAGCTGATGCTTTGGGCCGAGGACTGCCCCGAAGTCCGGCACCTCGTGCACGCGGATTTCGGCTCCAACAATGTCCTGACGGACAATGGCCGCATAACAGCGGTCATTGACTGGAGCGAGGCGATGTTCGGGGATTCCCAATACGAGGTCGCCAACATCTTCTTCTGGAGGCCGTGGTTGGCTTGTATGGAGCAGCAGACGCGCTACTTCGAGCGGAGGCATCCGGAGCTTGCAGGATCGCCACGACTCCGGGCGTATATGCTCCGCATTGGTCTTGACCAACTCTATCAGAGCTTGGTTGACGGCAATTTCGATGATGCAGCTTGGGCGCAGGGTCGATGCGACGCAATCGTCCGATCCGGAGCCGGGACTGTCGGGCGTACACAAATCGCCCGCAGAAGCGCGGCCGTCTGGACCGATGGCTGTGTAGAAGTACTCGCCGATAGTGGAAACCGACGCCCCAGCACTCGTCCGAGGGCAAAGAAAtagGGGCCCCGATCGTTCAAACATTTGGCAATAAAGTTTCTTAAGATTGAATCCTGTTGCCGGTCTTGCGATGATTATCATATAATTTCTGTTGAATTACGTTAAGCATGTAATAATTAACATGTAATGCATGACGTTATTTATGAGATGGGTTTTTATGATTAGAGTCCCGCAATTATACATTTAATACGCGATAGAAAACAAAATATAGCGCGCAAACTAGGATAAATTATCGCGCGCGGTGTCATCTATGTTACTAGATCAATTCAATTCGGCGTTAATTCAGTACATTAAAAACGTCCGCAATGTGTTATTAAGTTGTCTAAGCGTCAATTTGTTTACACCACAATATATCCTGCCA

RB, OsU6 promoter, sgRNA for SpCas12f, target sequence (OsTub-1), poly T, maize ubiqutin1 promoter, 5’ untranslated region of rice alcohol dehydrogenase, SV40 NLS, Rice codon-optimized SpCas12f, NOS terminator, 35S terminator, 35S promoter, hygromycin phosphotransferase, LB

**Supplementary Table S3. Mutation pattern results from amplicon sequence analysis**

Tub-1

| mutation pattern | experiment 1 | experiment 2 | experiment 3 | total | micro-homology | sequence |
| --- | --- | --- | --- | --- | --- | --- |
| WT |  |  |  |  |  | TTGGG**TTCACTGTGTACCCATCCCCTCA**GGTCTCCACCTCTGTGGTTGAGCCATACAACAGTGTCCTCTCTACC |
| -5 | 1 | 2 | 0 | 3 | TC-2 | TTGGG**TTCACTGTGTACCCATCCCCTC**-----TCCACCTCTGTGGTTGAGCCATACAACAGTGTCCTCTCTACC |
| -6-1 | 1 | 0 | 0 | 1 |  | TTGGG**TTCACTGTGTACCCATCCC**------TCTCCACCTCTGTGGTTGAGCCATACAACAGTGTCCTCTCTACC |
| -6-2 | 3 | 2 | 0 | 5 |  | TTGGG**TTCACTGTGTACCCATCCCC**------CTCCACCTCTGTGGTTGAGCCATACAACAGTGTCCTCTCTACC |
| -7-1 | 2 | 0 | 0 | 2 | CTC-1 | TTGGG**TTCACTGTGTACCCATCCCCTC**-------CACCTCTGTGGTTGAGCCATACAACAGTGTCCTCTCTACC |
| -7-2 | 0 | 3 | 1 | 4 | CTC-2 | TTGGG**TTCACTGTGTACCCATCCC**-------CTCCACCTCTGTGGTTGAGCCATACAACAGTGTCCTCTCTACC |
| -7-3 | 4 | 5 | 0 | 9 |  | TTGGG**TTCACTGTGTACCCATCC**-------TCTCCACCTCTGTGGTTGAGCCATACAACAGTGTCCTCTCTACC |
| -8-1 | 2 | 5 | 0 | 7 |  | TTGGG**TTCACTGTGTACCCATCCC**--------TCCACCTCTGTGGTTGAGCCATACAACAGTGTCCTCTCTACC |
| -8-2 | 6 | 2 | 0 | 8 |  | TTGGG**TTCACTGTGTACCCATC**--------TCTCCACCTCTGTGGTTGAGCCATACAACAGTGTCCTCTCTACC |
| -9-1 | 6 | 5 | 0 | 11 |  | TTGGG**TTCACTGTGTACCCATCC**---------TCCACCTCTGTGGTTGAGCCATACAACAGTGTCCTCTCTACC |
| -9-2 | 0 | 1 | 0 | 1 |  | TTGGG**TTCACTGTGTACCCAT**---------TCTCCACCTCTGTGGTTGAGCCATACAACAGTGTCCTCTCTACC |
| -10-1 | 12 | 9 | 0 | 21 | TC-1 | TTGGG**TTCACTGTGTACCCATC**----------TCCACCTCTGTGGTTGAGCCATACAACAGTGTCCTCTCTACC |
| -10-2 | 1 | 0 | 0 | 1 |  | TTGGG**TTCACTGTGTACCC**----------GTCTCCACCTCTGTGGTTGAGCCATACAACAGTGTCCTCTCTACC |
| -11-1 | 1 | 0 | 0 | 1 |  | TTGGG**TTCACTGTGTACCC**-----------TCTCCACCTCTGTGGTTGAGCCATACAACAGTGTCCTCTCTACC |
| -11-2 | 0 | 1 | 0 | 1 |  | TTGGG**TTCACTGTGTACCCA**-----------CTCCACCTCTGTGGTTGAGCCATACAACAGTGTCCTCTCTACC |
| -12-1 | 2 | 0 | 1 | 3 | TCC | TTGGG**TTCACTGTGTACCCATCC**------------ACCTCTGTGGTTGAGCCATACAACAGTGTCCTCTCTACC |
| -12-2 | 0 | 1 | 0 | 1 |  | TTGGG**TTCACTGTGTACCCATCCC**------------CCTCTGTGGTTGAGCCATACAACAGTGTCCTCTCTACC |
| -13 | 0 | 1 | 0 | 1 |  | TTGGG**TTCACTGTGTACCCATC**-------------ACCTCTGTGGTTGAGCCATACAACAGTGTCCTCTCTACC |
| -19 | 1 | 0 | 0 | 1 | CC-1 | TTGGG**TTCACTGTGTACCC**-------------------TCT**A**TGGTTGAGCCATACAACAGTGTCCTCTCTACC |
| -28-1 | 1 | 0 | 0 | 1 | CT | TTGGG**TTCACT**----------------------------CTGTGGTTGAGCCATACAACAGTGTCCTCTCTACC |
| -28-2 | 1 | 0 | 0 | 1 | TGT | TTGGG**TTCACTGTGT**----------------------------GGTTGAGCCATACAACAGTGTCCTCTCTACC |
| -30-1 | 0 | 2 | 0 | 2 | CTGTG | TTGGG**TTCACTGTG**------------------------------GTTGAGCCATACAACAGTGTCCTCTCTACC |
| -30-2 | 0 | 1 | 0 | 1 |  | TTGGG**TTC**------------------------------TCTGTGGTTGAGCCATACAACAGTGTCCTCTCTACC |
| -34 | 1 | 2 | 0 | 3 |  | TTGGG**TTCACTGTGTACCCA**----------------------------------ACAACAGTGTCCTCTCTACC |
| -42 | 1 | 0 | 0 | 1 | CC-2 | --------------------------------------TCTGTGGTTGAGCCATACAACAGTGTCCTCTCTACC |
| -49 | 1 | 0 | 0 | 1 | GTGT | TTGGG**TTCACTGTGT**-------------------------------------------------CCTCTCTACC |
| -82 | 0 | 1 | 0 | 1 | GCC | -------------------------------------------------------------------------- |
| G to T-1 | 0 | 8 | 1 | 9 |  | TTGGG**TTCACTGTGTACCCATCCCCTCA**G**T**TCTCCACCTCTGTGGTTGAGCCATACAACAGTGTCCTCTCTACC |
| G to T-2 | 0 | 2 | 0 | 2 |  | TTGGG**TTCACTGTGTACCCATCCCCTCAT**GTCTCCACCTCTGTGGTTGAGCCATACAACAGTGTCCTCTCTACC |
| A to C | 0 | 1 | 0 | 1 |  | TTGGG**TTCACTGTGTACCCATCCCCTCC**GGTCTCCACCTCTGTGGTTGAGCCATACAACAGTGTCCTCTCTACC |

Tub-2

| mutation pattern | experiment 1 | experiment 2 | experiment 3 | total | micro-homology | sequence |
| --- | --- | --- | --- | --- | --- | --- |
| WT |  |  |  |  |  | CACAA**TTCAAAAGACTGACCCACAATAA**ATAACTAGTCCTCAATTTAAAATTTGAGTTCCTAAATAGACATCTA |
| -2 | 0 | 1 | 0 | 1 |  | CACAA**TTCAAAAGACTGACCCACAA**--AATAACTAGTCCTCAATTTAAAATTTGAGTTCCTAAATAGACATCTA |
| -4-1 | 18 | 25 | 30 | 73 | ATAA | CACAA**TTCAAAAGACTGACCCACAATAA**----CTAGTCCTCAATTTAAAATTTGAGTTCCTAAATAGACATCTA |
| -4-2 | 0 | 1 | 0 | 1 |  | CACAA**TTCAAAAGACTGACCCA**----AAATAACTAGTCCTCAATTTAAAATTTGAGTTCCTAAATAGACATCTA |
| -5-1 | 0 | 2 | 0 | 2 |  | CACAA**TTCAAAAGACTGACCCACAA**-----AACTAGTCCTCAATTTAAAATTTGAGTTCCTAAATAGACATCTA |
| -5-2 | 0 | 0 | 1 | 1 |  | CACAA**TTCAAAAGACTGACCCACAATA**-----CTAGTCCTCAATTTAAAATTTGAGTTCCTAAATAGACATCTA |
| -6-1 | 3 | 2 | 1 | 6 |  | CACAA**TTCAAAAGACTGACCCACAA**------ACTAGTCCTCAATTTAAAATTTGAGTTCCTAAATAGACATCTA |
| -6-2 | 0 | 1 | 1 | 2 |  | CACAA**TTCAAAAGACTGACCCAC**------TAACTAGTCCTCAATTTAAAATTTGAGTTCCTAAATAGACATCTA |
| -7-1 | 8 | 8 | 10 | 26 | AA | CACAA**TTCAAAAGACTGACCCACAA**-------CTAGTCCTCAATTTAAAATTTGAGTTCCTAAATAGACATCTA |
| -7-2 | 3 | 2 | 1 | 6 |  | CACAA**TTCAAAAGACTGACCCA**-------TAACTAGTCCTCAATTTAAAATTTGAGTTCCTAAATAGACATCTA |
| -8-1 | 0 | 1 | 0 | 1 | TA | CACAA**TTCAAAAGACTGACCCACAATA**--------GTCCTCAATTTAAAATTTGAGTTCCTAAATAGACATCTA |
| -8-2 | 5 | 3 | 1 | 9 |  | CACAA**TTCAAAAGACTGACCCACA**--------CTAGTCCTCAATTTAAAATTTGAGTTCCTAAATAGACATCTA |
| -8-3 | 1 | 0 | 0 | 1 |  | CACAA**TTCAAAAGACTGACCCA**--------AACTAGTCCTCAATTTAAAATTTGAGTTCCTAAATAGACATCTA |
| -9-1 | 6 | 1 | 2 | 9 |  | CACAA**TTCAAAAGACTGACCCA**---------ACTAGTCCTCAATTTAAAATTTGAGTTCCTAAATAGACATCTA |
| -9-2 | 1 | 2 | 1 | 4 |  | CACAA**TTCAAAAGACTGACCCAC**---------CTAGTCCTCAATTTAAAATTTGAGTTCCTAAATAGACATCTA |
| -9-3 | 0 | 0 | 1 | 1 |  | CACAA**TTCAAAAGACTGACCCACA**---------TAGTCCTCAATTTAAAATTTGAGTTCCTAAATAGACATCTA |
| -10-1 | 9 | 7 | 11 | 27 | AC | CACAA**TTCAAAAGACTGACCCAC**----------TAGTCCTCAATTTAAAATTTGAGTTCCTAAATAGACATCTA |
| -10-2 | 0 | 1 | 0 | 1 |  | CACAA**TTCAAAAGACTGACCCACAA**----------GTCCTCAATTTAAAATTTGAGTTCCTAAATAGACATCTA |
| -11-1 | 1 | 1 | 0 | 2 |  | CACAA**TTCAAAAGACTGACCCA**-----------TAGTCCTCAATTTAAAATTTGAGTTCCTAAATAGACATCTA |
| -11-2 | 0 | 1 | 0 | 1 |  | CACAA**TTCAAAAGACTGACCC**-----------CTAGTCCTCAATTTAAAATTTGAGTTCCTAAATAGACATCTA |
| -11-3 | 0 | 1 | 0 | 1 |  | CACAA**TTCAAAAGACTGACCCACAAT**-----------CCTCAATTTAAAATTTGAGTTCCTAAATAGACATCTA |
| -12-1 | 2 | 2 | 0 | 4 |  | CACAA**TTCAAAAGACTGACCC**------------TAGTCCTCAATTTAAAATTTGAGTTCCTAAATAGACATCTA |
| -12-2 | 0 | 1 | 0 | 1 |  | CACAA**TTCAAAAGACTGA**------------AACTAGTCCTCAATTTAAAATTTGAGTTCCTAAATAGACATCTA |
| -13 | 0 | 1 | 0 | 1 |  | CACAA**TTCAAAAGACTGACCCA**-------------GTCCTCAATTTAAAATTTGAGTTCCTAAATAGACATCTA |
| -18 | 0 | 1 | 0 | 1 | ACT | CACAA**TTCAAAAGACT**------------------AGTCCTCAATTTAAAATTTGAGTTCCTAAATAGACATCTA |
| -19 | 0 | 0 | 1 | 1 |  | CACAA**TTCAAAAGACTG**-------------------TCCTCAATTTAAAATTTGAGTTCCTAAATAGACATCTA |
| -22 | 0 | 1 | 0 | 1 |  | CACAA**TTCAAA**----------------------TAGTCCTCAATTTAAAATTTGAGTTCCTAAATAGACATCTA |
| -23 | 1 | 0 | 0 | 1 |  | CACAA**TTCAAAAGACTGACCCACAATAA**-----------------------TTGAGTTCCTAAATAGACATCTA |
| +1 | 1 | 0 | 0 | 1 |  | CACAA**TTCAAAAGACTGACCCACAATAA**ATAA**A**CTAGTCCTCAATTTAAAATTTGAGTTCCTAAATAGACATCTA |
| +2, A to T, T to A, C to T | 0 | 1 | 0 | 1 |  | CACAA**TTCAAAAGACTGACCCACAATTT**A**A**AA**T**T**TG**AGTCCTCAATTTAAAATTTGAGTTCCTAAATAGACATCTA |

**Supplementary Table S4. Off-target analysis in T_1_ plants**

| Tub-1 #12 | **Target** | **Off-target** |
| --- | --- | --- |
|  | mutated/analyzed colony | mutated/analyzed colony |
| 1 (WT) | 0/10 | 0/6 |
| 2 (homo) | 7/7 | 0/11 |
| 3 (homo) | 8/8 | 0/8 |
| 4 (hetero) | 1/6 | 0/11 |
| 5 (WT) | 0/10 | 0/8 |
| 6 (hetero) | 6/9 | 0/8 |
| 7 (hetero) | 5/7 | 0/8 |
| 8 (hetero) | 2/4 | 0/13 |
| 9 (homo) | 10/10 | 0/6 |
| 10 (WT) | 0/8 | 0/6 |

Off-target analysis was conducted by performing PCR using primers capable of amplifying both the target region and the off-target region and performing Sanger sequencing of colonies obtained by cloning the PCR product into *E. coli*.

**Supplementary Figure S1. Electrophoresis data of HMA in *OsTubulin* locus**

Tub-1

Tub-2

The numbers 1–10 and 11–20 indicate the pick-up sample in Figure2A. NT; non-transformed, -g; calli transformed with pPZP-SpCas12f vector without sgRNA.

**Supplementary Figure S2. Electrophoresis data of HMA and sequence analysis in *OsHDAC1* locus**

| WT |  | CGAG**TTCCCCCCGCCTCCGGCGGAGGC**GTCCATGGCGGCG | (%) |
| --- | --- | --- | --- |
| HDAC #1 | -3 bp | CGAG**TTCCCCCCGCCTCCGGCGGAGGC**GTCCAT---GGCG | 5.1 |

Details are the same as in Figure 2B.

**Supplementary Figure S3. Photos of T_1_ plants**

Tub-1 #12

WT

homo

homo

WT

**
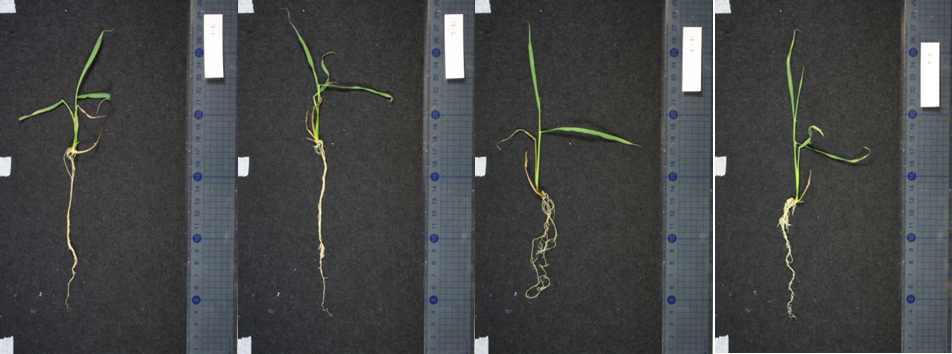
**

Tub-2 #15-3

WT

homo

homo

WT


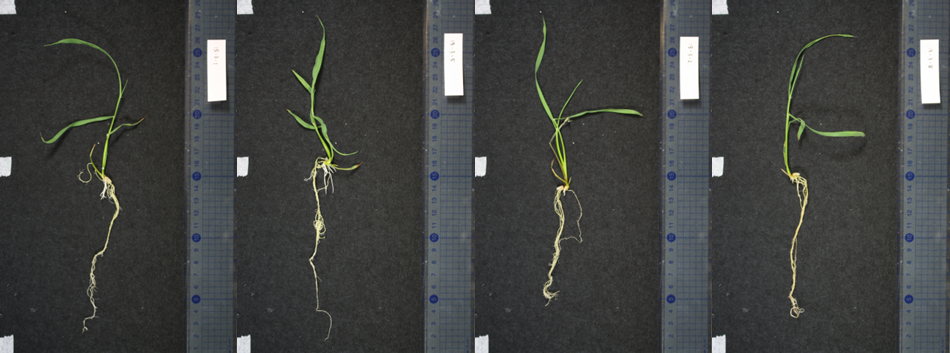

Supplement: Supplementary file 1 [file Table1.DOCX]
